# Genome sequence data of the plant growth-promoting rhizobacterium *Pseudomonas inefficax* MG-2

**DOI:** 10.1128/mra.00321-26

**Published:** 2026-05-06

**Authors:** Marat Lutfullin, Daria Pudova, Leyla Minnullina, Anastasiia Nikolaeva, Guzel Lutfullina, Yaw Akosah, Elena Shagimardanova, Ayslu Mardanova

**Affiliations:** 1 Department of Microbiology, Institute of Fundamental Medicine and Biology, Federal University 64922, Kazan, Russia; 2 Laboratory of Agrobioengineering, Institute of Fundamental Medicine and Biology, Federal University 64922, Kazan, Russia; 3 Department of Molecular Pathobiology, New York University College of Dentistry 70241, New York, New York, USA; 4 Loginov Moscow Clinical Scientific Center 431062 , Moscow, Russia; University of Southern California, Los Angeles, California, USA

**Keywords:** genome sequence, *Pseudomonas inefficax* MG-2, rhizobacteria

## Abstract

We present the findings from the genome sequencing project of a plant growth-promoting rhizobacterium *Pseudomonas inefficax* MG-2, sourced from rhizospheric soil. The genome spans 5,736,804 base pairs with an average GC content of 62.9%. An incomplete plasmid with a length of 16,324 base pairs and a GC content of 52.6% was also identified.

## ANNOUNCEMENT

Plant growth-promoting rhizobacteria (PGPR) enhance plant development via direct and indirect pathways, facilitating nutrient acquisition and abiotic stress tolerance through phytohormone production and ACC deaminase synthesis (1–3). We investigated PGPR strain *Pseudomonas inefficax* MG-2, isolated from the “Zhukovskij rannij” potato rhizosphere (GPS: N55.615850°, E49.335570°). This strain produces indole-3-acetic acid and siderophores, enhancing germination and growth of rye, pea, wheat, and potato (4). Whole-genome sequencing identified genetic loci responsible for its secondary metabolite biosynthesis.

To isolate the bacterium, non-rhizosphere soil was removed by shaking. Roots (5 g) were placed in phosphate-buffered saline (50 mL). Dilutions of the suspension were plated on Tryptic soy agar (15.0 g casein peptone [pancreatic], 5.0 g soya peptone [papainic], 5.0 g NaCl, 15 g agar; 1,000 mL), and incubated for 24 hours at 30°C (5). A single colony was picked for isolation. Pure culture was grown in Tryptic soy broth at 30°C overnight (180 rpm). Genomic DNA from *P. inefficax* MG-2 was extracted using the NucleoSpin Microbial DNA Extraction Kit.

DNA concentration was measured with a NanoDrop 2000c (Thermo Scientific), yielding 500 ng/µL. DNA was fragmented using Covaris S220, and libraries were prepared with the NEBNext Ultra II Kit (New England Biolabs). Quality was verified via 2100 Bioanalyzer (Agilent Technologies) and a high-sensitivity DNA kit (Agilent Technologies). DNA sequencing was executed on the Illumina MiSeq platform (Illumina) with a 250 bp paired-end library. Sequencing data were evaluated using FastQC v0.12.1 software (6). Adapter trimming and quality filtering were performed using Trimmomatic v0.39 (7) (ILLUMINACLIP:TruSeq3-PE.fa:2:30:10, SLIDINGWINDOW:4:20, MINLEN:30). As a result, 8,493,454 raw reads were obtained, which were used for *de novo* genome assembly by SPAdes v3.15.4 (8) with the “*--careful*” parameter. The genome assembly comprised 160 contigs. QUAST v5.2.0 (9) analysis showed that after removing short sequences with low coverage, 32 contigs (≥500 bp, ≥2.0 coverage) remained, which constituted a genome with a total length of 5,736,804 bp, GC content of 62.9%, N50 value of 421,001 bp, L50 value of 5 contigs, and coverage of 127×. An incomplete 16.3 Kb plasmid (52.6% GC), homologous to *Pseudomonas fluorescens* pPHE69 (MH061179), was identified via mlplasmids v2.1.0 (10) and geNomad v1.9 (11).

ANIb analysis using JSpeciesWS (12) against 20 related *Pseudomonas* genomes showed 99.13% identity with *P. inefficax* 17-08R strain (NZ_JBHGVU000000000). NCBI Prokaryotic Genome Annotation Pipeline v6.10 annotation identified 5,274 genes (5,125 CDS, 67 tRNA, 8 rRNA, and 70 pseudogenes). Analysis of the annotated genome revealed auxin biosynthesis clusters, including aromatic-L-amino-acid decarboxylase (EC 4.1.1.28), indole-3-glycerol phosphate synthase, and tryptophan synthase (EC 4.2.1.20). Siderophore biosynthesis genes were identified: TonB-dependent ferric achromobactin receptor protein, aerobactin siderophore receptor *iutA*, and iron siderophore receptor protein.

Using antiSMASH 8.0.4 (13), gene clusters associated with secondary metabolites, such as O-antigen, pseudopyronine A/B, grimoviridin, lipopolysaccharide, azotobactin D, and gamexpeptide A/B/E or luminmide B/D/E/F/G, were identified in the *P. inefficax* MG-2 genome. Default parameters were used for all software unless otherwise specified.

Thus, whole-genome sequencing of *P. inefficax* MG-2 reveals the underlying genetic mechanisms responsible for its growth-stimulating properties.

## Data Availability

The data of the whole genome shotgun sequencing project were uploaded to DDBJ/EMBL/GenBank under accession number JAHHZI000000000, BioProject accession number PRJNA732441, and BioSample accession number SAMN19320528. The raw reads were deposited in the SRA database under the corresponding BioProject accession number.
